# Recombination: the good, the bad and the variable

**DOI:** 10.1098/rstb.2017.0279

**Published:** 2017-11-06

**Authors:** Jessica Stapley, Philine G. D. Feulner, Susan E. Johnston, Anna W. Santure, Carole M. Smadja

**Affiliations:** 1 Centre for Adaptation to a Changing Environment, IBZ, ETH Zürich, Zürich, Switzerland; 2 Department of Fish Ecology and Evolution, Centre of Ecology, Evolution and Biogeochemistry, EAWAG Swiss Federal Institute of Aquatic Science and Technology, 6047 Kastanienbaum, Switzerland; 3 Division of Aquatic Ecology and Evolution, Institute of Ecology and Evolution, University of Bern, 3012 Bern, Switzerland; 4 Institute of Evolutionary Biology, University of Edinburgh, Edinburgh EH9 3JY, UK; 5 School of Biological Sciences, University of Auckland, Auckland 1142, New Zealand; 6 Institut des Sciences de l'Evolution UMR 5554, CNRS, IRD, EPHE, Université de Montpellier, 3095 Montpellier cedex 05, France

**Keywords:** crossing over, meiosis, genetic linkage, evolution, adaptation, genomics

## Abstract

Recombination, the process by which DNA strands are broken and repaired, producing new combinations of alleles, occurs in nearly all multicellular organisms and has important implications for many evolutionary processes. The effects of recombination can be *good*, as it can facilitate adaptation, but also *bad* when it breaks apart beneficial combinations of alleles, and recombination is highly *variable* between taxa, species, individuals and across the genome. Understanding how and why recombination rate varies is a major challenge in biology. Most theoretical and empirical work has been devoted to understanding the role of recombination in the evolution of sex—comparing between sexual and asexual species or populations. How recombination rate evolves and what impact this has on evolutionary processes within sexually reproducing organisms has received much less attention. This Theme Issue focusses on how and why recombination rate varies in sexual species, and aims to coalesce knowledge of the molecular mechanisms governing recombination with our understanding of the evolutionary processes driving variation in recombination within and between species. By integrating these fields, we can identify important knowledge gaps and areas for future research, and pave the way for a more comprehensive understanding of how and why recombination rate varies.

## 1. Introduction

Recombination, the exchange of DNA between maternal and paternal chromosomes during meiosis, is a near universal processes occurring in almost all forms of life and is fundamental for DNA repair and meiotic cell division. Recombination is *good* as it can facilitate adaptation through the creation of novel genetic combinations [1,2], but also *bad* as it can break apart favourable combinations of alleles [3], and despite meiosis and recombination being highly regulated, recombination is frequently *variable* across the genome, across taxa, between the sexes, populations and individuals [4]. Although ongoing advances in DNA sequencing technology and methods to estimate recombination from population-based samples are providing much needed empirical evidence of ‘How’ recombination varies, our understanding of ‘Why’ it varies is progressing more slowly.

When considering this question: ‘Why does recombination rate vary?’, there is much focus on the evolutionary advantage of sex, but much less attention given to understanding why recombination varies between sexually reproducing organisms. For us, as a group of evolutionary biologists who have observed variation in recombination rate in sexually reproducing organisms—we considered this an important knowledge gap. Hence, we proposed this special issue ‘Evolutionary causes and consequences of recombination rate variation in sexual organisms' with the aim to bring this question to the fore and to encourage more researchers to investigate if and how recombination rate varies, whether it responds to natural or sexual selection, and how it influences fundamental evolutionary processes within sexually reproducing organisms, such as adaptation and speciation.

To understand the evolution of recombination, like any trait, we need information on the heritability and plasticity of the trait, on how the trait influences fitness, and on the molecular mechanisms controlling the trait. In this respect, we know a lot about recombination—recombination is heritable, many populations harbour additive genetic variation for it, and it can respond to selection in the laboratory [4]. Recombination is also modified by a range of environmental stimuli, including temperature [5] and condition [6]. Recombination has well-characterized fitness effects: for example, in humans, altered rates of recombination can cause chromosomal abnormalities, reduced fertility and disease [7]. Enormous progress has been made recently in understanding the genetic elements that control recombination, and these include elements that are highly conserved across eukaryotes (e.g. SPO11), as well as evolutionarily dynamic elements such as PRDM9 [8]. However, unlike other traits that evolutionary biologists commonly study, recombination has some very unusual properties: it can influence the efficacy of selection [9,10], facilitate adaptation to changing environments [1,11], it can alter patterns of nucleotide diversity [8] and influence genetic diversity within populations [12,13]. Thus, variation in recombination can experience direct and indirect selection, and this selection can result from short-term (in the next generation) and long-term (over many generations) benefits.

Addressing the question ‘Why does recombination rate vary?’ necessitates understanding both direct and indirect effects of selection. However, most researchers often do not consider selection operating at these different scales simultaneously. For example, population geneticists often view recombination as a population parameter that is under indirect and long-term selection, whereas developmental biologists consider the direct, short-term effects that an altered rate of recombination has on individual traits like fertility. One goal of this Special Issue is to try to better integrate these different perspectives. Thus, we sought contributions from the ‘population genetics–evolutionary’ perspective [14–16] as well as the ‘developmental–molecular’ perspective [5,7,8], with the hope that this will foster greater communication across these disciplines.

The Special Issue opens with a general review of how and why recombination rate varies across eukaryotes. It includes an analysis of data from the largest collection of linkage maps to date and an overview of the processes that explain variation in recombination rate—from genome architecture to evolutionary explanations. The review introduces many of the concepts and evolutionary theories that are explored in greater depth in the proceeding articles. Although empirical data for recombination are growing rapidly, progress in explaining this variation has been slow. Dapper & Payseur [17] argue that progress in the field is hampered by a disconnect between empirical data and the large body of theory that has been developed to explain variation in recombination. For example, much theory has been developed to explain the evolutionary advantage of recombination, but this theory does not address quantitative differences among individuals or variation at different genomic scales [17]. Likewise, empirical data are often not collected to directly test current theories and to address this Dapper & Payseur [17] provide a useful table that lists the main hypotheses proposed to explain the evolution of recombination, along with their key requirements and testable predictions relating to each.

Variation in recombination may be explained by variation in the sexual system and the evolutionary consequences of different reproductive modes. For example, selfing and inbreeding species may benefit from high rates of recombination because it can increase genetic diversity and allelic shuffling, and there is some evidence to support this [4]. However, the transition to obligate asexuality may result in suppression of recombination, because asexuals often have modified meiosis and because recombination can erode heterozygosity in asexuals [18]. To investigate how recombination rate varies between sexual and asexual species, Haag *et al*. [18] build a new linkage map for an undescribed species of brine shrimp whose closest relative is an obligate asexual. In their focal sexual species, they report one of the shortest linkage maps known, and propose that the observed low recombination rates in some sexual species may favour sex–asex transitions or, alternatively, may be a consequence of it. The generality of this finding has yet to be tested and Haag *et al*. [18] acknowledge that counter examples in other groups exist. One notable counter example is observed in *Daphnia*, a genus that contains both sexual and asexual parthenogenetic species. In cyclically parthenogenic species for which linkage maps have been made, we observe a very high recombination rate per megabase compared to other crustaceans [4]. These apparent contradictory results demonstrate that we need more recombination data in a greater ranges of species in order to begin to address long-standing theories on the evolution of recombination.

A notable pattern to emerge from empirical data is the observation that the distribution of recombination events is distinctly non-random across the genome. Variation is observed at many genomic scales: between chromosomes, between megabase regions within chromosomes and across regions spanning only a few kilobases. Between closely related species, there is often more variation in the distribution of recombination events compared to the total frequency of these events, suggesting that the rate at which recombination evolves is likely to depend on the genomic scale [4,17]. Variable rates of recombination between chromosomes have long been recognized, with sex chromosomes representing the most well-known example of this phenomenon as non-recombining sex chromosomes have evolved multiple times across taxa. The repeatability with which sex chromosomes have evolved suppressed recombination makes them excellent models to investigate how selection drives regional suppression of recombination and to identify proximate mechanisms [16]. In her contribution, Charlesworth [16] reviews the proximate and ultimate mechanisms driving suppression of recombination in sex chromosomes and provides recommendations of how to study this in divergent systems that differ in the age of their sex-linked regions.

Estimates of recombination at the fine genomic scale can provide information about how recombination influences or is influenced by genetic elements and neighbouring DNA sequence. Recombination is often higher in sequence regions with high gene density and GC content and lower in sequence regions that are enriched for simple repeats and transposable elements (TE). Although these patterns are known from a wide range of eukaryotes, the proximate and ultimate mechanisms driving these correlations are poorly understood. To begin to address this problem, Kent *et al*. [19] provide a comprehensive review of how and why recombination rate is correlated with TE density. In their review, they strive to address the problem of ‘cause and effect’ in this relationship, which is challenging because TE density can be modified by local recombination rate (i.e. TEs accumulate in regions of low recombination), but TEs can also modify the local recombination rate when the host genomes epigenetically silence TEs, and this often results in suppressed recombination [19]. Kent *et al*. [19] propose that the relationship between TEs and recombination may be better understood as ‘coevolution’, which involves positive feedback between recombination suppression and TE accumulation.

Another commonly observed pattern in sequence data is a positive correlation between recombination rate and nucleotide diversity. This relationship can be driven by the mutagenic effects of recombination [8], but it can also be the result of purifying selection acting on a deleterious mutation, and in doing so reducing genetic diversity at neighbouring, genetically liked loci (a process referred to as background selection (BGS)) [12]. This size of the region that experiences a loss in diversity is determined by the strength of selection (increasing with increasing selection) and the local recombination rate (larger in regions of low recombination). Comeron [12] reviews the concepts behind BGS, and argues that studies that attempt to investigate evidence for selection in the genome using patterns of nucleotide diversity should use a null model that incorporates BGS. With this approach, we can better determine baseline levels of diversity across the genome and identify the regions experiencing different forms of selection [12].

A pervasive pattern to emerge from accumulating fine-scale recombination data is that in many species, recombination is localized to small genomic regions referred to as hotspots. Hotspots are present in a wide range of species, but absent from some well-known model species such as *Caenorhabditis elegans* [20] and *Drosophila* [21]. Progress in understanding the molecular mechanisms controlling hotspot position and activity has fuelled exciting empirical and theoretical work. Tiemann-Boege *et al*. [8] provide a comprehensive review of recombination hotspots and the processes governing double-strand breaks (DSBs), which precede recombination. Repair of DSBs often introduces changes into the sequence—either via mutations or via biased gene conversion, and in many cases, the allele that initiates the DSB (active allele) is converted to an inactive allele—turning hotspots cold [8,15]. One mechanism controlling hotspot activity in several mammals is PRDM9, a zinc-finger binding protein that recognizes and binds to sequence motifs, initiating a DSB. Investigation of this *trans*-acting factor has provided a great deal of insight into how and why hotspot activity varies between species [22]. The rapid turnover of hotspots controlled by PRDM9 can also drive reproductive isolation between mouse strains [8] and PRDM9 was originally described as a speciation gene [7]. The self-destructive nature of PRDM9-directed hotspots creates rapid evolutionary dynamics—when an active allele is converted to an inactive allele and recombination rate declines, selection will favour new PRDM9 alleles in order to restore the optimal level of recombination for the species [15]. To model these rapid dynamics, Latrille *et al*. [15] develop a population-genetic Red Queen model, explore the behaviour of their model over a range of conditions via simulations, and finally support their model with analytical and numerical approximations [15]. Importantly, their analyses demonstrate that for low-scaled mutation rates (i.e. the mutation rate multiplied by the effective population size), there tends to be only one PRDM9 allele that dominates, which is then replaced through hard sweeps as the recombination targets in the genome are eroded. By contrast, at higher scaled mutation rates, a population of PRDM9 alleles can exist, each binding unique motifs in the genome [15].

Variation in recombination is not solely due to genetic factors: a multitude of environmental factors both extrinsic and intrinsic have been found to alter recombination frequency and distribution. Experimental work in *Drosophila melanogaster* has been instrumental in the development of this field and Stevison *et al*. [6] review this body of work and explore the possible molecular mechanisms governing plasticity. The authors also reflect on the lessons learnt and provide recommendations for future empirical research, which they successfully implement in a related species *D. pseudoobscura* as a proof of concept and to provide new data in less characterized species [6]. Alves *et al*. [7] explore the vulnerabilities of human meiosis, one of which is maternal age, an intrinsic factor that is often associated with a greater frequency of chromosomal abnormalities and altered recombination. One hypothesis proposed to explain increased aneuploidy in older females is that recombination frequency or distribution changes with age; however, Alves and coauthors argue that this is unlikely because the effects of maternal age on recombination are not consistent or strong enough to drive the patterns of aneuploidy observed [7].

While there are many environmental cues known to influence recombination, temperature and condition are perhaps the most well known. Morgan *et al*. [5] provide a comprehensive review of how temperature can influence meiosis and thus recombination through changes in the axes, which unite sister chromatids, and the synaptonemal complex, the protein structure that forms between these chromatids during meiosis. As proteins responsible for the formation of those structures during meiosis are in general (across eukaryotes) prone to aggregation and hence are particularly temperature-sensitive, this could explain why temperature often has a relatively consistent effect on recombination across taxa [5]. The second important environmental cue to influence recombination is condition. An organism in poor condition or in a poor environment may benefit from shuffling its genome, as its haplotype is poorly matched to the current environment, and there is some empirical evidence to support this [14]. Previous theoretical work suggests that condition-dependent recombination seems to emerge most readily in models of haploid rather than diploid selection, and that recombination rate plasticity is unlikely unless, for example, maternal effects are assumed. Rybnikov *et al*. [14] construct a simulation model to test the emergence of condition-dependent recombination rate. Their model demonstrates that in the case where recombination rates *within* a group of selected loci are determined by an unlinked locus, alleles at the locus conferring condition-dependent recombination can readily invade a population with a fixed recombination rate. These modelling results agree with theoretical studies that indicate that in diploids, direct effects—where the locus determining recombination influences recombination *between* itself and the group of selected loci—are unlikely to lead to plasticity in recombination rate. Interestingly, the simulations in the paper also demonstrate that plasticity in recombination rate requires that the population spends relatively equal time in different selective environments—if the majority of time is spent in a single environment, a single, fixed rate of recombination is favoured [4].

In conclusion, in spite of exciting recent results, understanding how and why recombination rate varies across the genome and across taxa remains a major challenge in biology. For this reason, we present a series of contributions that critically evaluate theoretical and empirical developments on variation in recombination rate between taxa [4,17,18] and across the genome [7,8,12,16,19], and outline some tentative explanations, both mechanistic [5] and evolutionary [6,14,15], for this variation. This Special Issue includes work from scholars who differ in their perspective, but whose approaches and study systems are complementary. Such integration has not been attempted before and it is our hope that this theme issue will contribute to a comprehensive conceptual framework that will inspire a wider range of theoretical and empirical tests for the evolution of recombination rate—covering the *good*, the *bad* and the *variable*.

## Data accessibility

This article has no additional data.
